# Knockdown of CXCL1 improves ACLF by reducing neutrophil recruitment to attenuate ROS production and hepatocyte apoptosis

**DOI:** 10.1097/HC9.0000000000000257

**Published:** 2023-09-15

**Authors:** Shima Tang, Junlei Zhang, Lingjian Zhang, Yalei Zhao, Lanlan Xiao, Fen Zhang, Qian Li, Ya Yang, Qiuhong Liu, Jinxian Xu, Lanjuan Li

**Affiliations:** 1 State Key Laboratory for Diagnosis and Treatment of Infectious Diseases, National Clinical Research Center for Infectious Diseases, Collaborative Innovation Center for Diagnosis and Treatment of Infectious Diseases, The First Affiliated Hospital, Zhejiang University School of Medicine, Hangzhou, China; 2 Zhejiang Provincial Key Laboratory of Pancreatic Disease, the First Affiliated Hospital, Zhejiang University School of Medicine, Hangzhou, China; 3 Department of Infectious Diseases, The Affiliated Hangzhou First People’s Hospital, School of Medicine, Zhejiang University, Hangzhou, China; 4 Department of Infectious Diseases, The First Affiliated Hospital of Xi’an Jiaotong University, Xi’an, China

## Abstract

**Background:**

Acute-on-chronic liver failure (ACLF) is an acute decompensated syndrome based on chronic liver disease, while neutrophil recruitment is the most critical early step. C-X-C motif chemokine ligand 1 (CXCL1), a cytokine that recruits neutrophils, was significantly upregulated in both ACLF mice and patients with ACLF. This present study aims to explore the role of CXCL1 in the pathogenesis of ACLF.

**Methods:**

We established an ACLF mouse model induced by carbon tetrachloride, lipopolysaccharide, and D-galactosamine, and used adeno-associated virus to achieve overexpression and knockdown of *Cxcl1*. We employed mass cytometry, flow cytometry, multiplex cytokine and chemokine analysis, Western blot, and reactive oxygen species (ROS) detection in mice blood and liver. ACLF patients (n = 10) and healthy controls (n = 5) were included, and their liver samples were stained using multiplex immunohistochemistry techniques.

**Results:**

CXCL1 was significantly elevated in both ACLF mice and patients. CXCL1 recruits neutrophils by binding to the C-X-C motif chemokine receptor 2 on the surface of neutrophils, affects ACLF prognosis by generating ROS and mitochondrial depolarization and modulating caspase3-related apoptotic pathways. We found that the knockdown of CXCL1 attenuated the infiltration of neutrophils in the mouse liver, reduced the expression of inflammatory cytokines, and also significantly downregulated ROS production and caspase3-related hepatocyte apoptosis, thereby ameliorating the liver injury of ACLF.

**Conclusions:**

CXCL1 is a core player in the mobilization of neutrophils in ACLF, and the knockdown of Cxcl1 improves neutrophil infiltration, reduces ROS levels, and reduces hepatocyte apoptosis, thereby attenuating inflammation and liver injury in ACLF. Our results revealed a previously unknown link between CXCL1-induced neutrophil recruitment and ACLF, providing evidencing for potential therapies targeting ACLF.

## INTRODUCTION

Acute-on-chronic liver failure (ACLF) is a syndrome of acute decompensation based on chronic liver disease, accompanied by high short-term mortality and multiple organ failure.^1^ In Western countries, ACLF is more common in young patients with alcohol-associated cirrhosis, whereas in the Asia-Pacific region, the main cause is HBV infection, with ~70% of ACLF cases being HBV-ACLF.^2^ Currently, there is no effective treatment for ACLF. Liver transplantation is the only cure.^3^ However, the number of liver donors is far less than the number of patients. Thus, ACLF urgently needs new early warning and treatment methods.

As the first barrier of the innate immune system, neutrophils play a vital role in the development of liver injury, including alcohol-associated hepatitis, acute liver failure, and ACLF.^4–8^ A study by Casulleras et al showed that patients with ACLF had significantly higher neutrophil counts compared with those of healthy individuals and patients with cirrhosis.^9^ Following bacterial infection and/or tissue injury, neutrophils are rapidly activated, move out of blood vessels, and migrate along a concentration gradient of chemoattractants to target sites, where they recognize and bind to pathogens through pathogen-associated molecular patterns, resulting in phagocytosis of the pathogens.^10^ During phagocytosis, the NADPH oxidase complex is activated and releases antimicrobial peptides, proteases, and numerous reactive oxygen species (ROS) to kill and destroy bacteria.^11^ ROS are normal by-products of aerobic metabolism and are produced in mitochondria; however, excess ROS can cause oxidative damage to cells, resulting in intracellular oxidative stress and cell damage. The liberation of cellular contents further exacerbates inflammatory damage. In addition, ROS also promotes the secretion of cytokines, leading to an increase in ROS production, forming a vicious circle and promoting the deterioration of liver disease.

Several signaling pathways are involved in the chemotaxis and recruitment of neutrophils, and one of the important pathways is the C-X-C motif chemokine ligand 1 (CXCL1)-C-X-C motif chemokine receptor 2 (CXCR2) pathway. CXCL1, a member of the CXC chemokine family, mediates neutrophil recruitment by binding to CXCR2 and is important for neutrophil activation, recruitment, and infiltration.^12^ CXCL1 is highly expressed during the inflammatory response, thereby promoting the inflammatory process. It plays an important role in neutrophil-dependent immune-induced lung inflammation, sepsis, hepatitis, and HSV1-related encephalitis.^13–15^ In addition to inducing inflammation, recent studies have demonstrated that CXCL1 is involved in the pathogenesis of liver diseases, such as NASH, HCV-related hepatitis, DILI, and HCC.^16–18^ Chang et al demonstrated that blocking CXCL1 reduced hepatic neutrophil infiltration and injury in a mouse model of NASH, whereas *Cxcl1* overexpression exacerbated steatohepatitis in mice.^19^ A study by Hilscher et al showed that the upregulation of CXCL1 promotes neutrophil aggregation and induces hepatic sinusoidal thrombosis and fibrosis through the formation of neuroendocrine tumors.^20^

Previously, in a prospective study, we reported that serum CXCL1 levels and neutrophil counts were significantly higher in patients with HBV-ACLF than in healthy controls and chronic HBV groups and that serum CXCL1 was an independent risk factor for HBV-ACLF prognosis.^21^ However, the unresolved challenge is that we do not know the pathway through which CXCL1 affects the progression of ACLF. In the present study, we established an ACLF mouse model, achieved the overexpression and knockdown of the *Cxcl1* gene by adeno-associated virus infection, and confirmed that CXCL1 can recruit neutrophils in the liver. We explored the potential mechanism of CXCL1 affecting the process of ACLF and found that it may trigger some signals that lead to massive death of liver cells, such as excessive production of inflammatory factors and ROS, mitochondrial dysfunction, etc., inducing a large number of liver cell apoptosis, and cause damage to the liver. In addition, we performed further validation in human liver tissue using multiplex immunofluorescence techniques and found that patients with ACLF with low CXCL1 expression had lower levels of neutrophils and ROS in their liver tissue.

## METHODS

### Patients

Liver samples for this study were obtained from 5 healthy donors and 10 patients with ACLF who underwent liver transplantation. The protocol was approved by the Human Ethics Committee of the First Affiliated Hospital of Zhejiang University School of Medicine, and written informed consent was obtained from the subjects (license number: IIT20220273B).

### Mice

Wild-type male C57BL/6 mice, 6–8 weeks old, were obtained from the Animal Experiment Center of Zhejiang Academy of Medical Sciences. They were raised in a room with standard temperature and light, with free access to adequate food and water. All animals were cared for in accordance with the guidelines of the Zhejiang Provincial Institute of Health. Our animal experiment protocol was approved by the Animal Care Committee of the First Affiliated Hospital of Zhejiang University School of Medicine for Animal Experiment Ethics Examination (license number: 2022-1617).

### Model of ACLF

To mimic the clinical progression of ACLF, we combined long-term CCl_4_ injection with a single dose of LPS/D-GaIN. Mice were randomly divided into 4 groups: NC group, CCl4 group, D-Gal + LPS group, and ACLF group. The NC group (n = 10) was continuously injected with olive oil for 8 weeks, sacrificed after the last injection, and the serum and liver tissue were collected. Group CCl4 (n = 10) was i.p. injected with a mixture of CCl4 (0.6 ml/kg, Aladdin, Shanghai, China) and olive oil twice a week for 8 weeks and then sacrificed. Group D-Gal + LPS (n = 10) received a single i.p. injection of D-GalN (1000 mg/kg, Sigma, St. Louis, MO, USA) and LPS (100 ng/kg, Sigma) and then sacrificed. The mice in group ACLF were i.p. injected with a mixture of CCl_4_ (0.6 ml/kg, Aladdin, Shanghai, China) and olive oil for 8 weeks. At 24 hours after the last injection, they were i.p. injected with D-GalN (1000 mg/kg, Sigma, St. Louis, MO, USA) and LPS (100 ng/kg, Sigma), and their survival was observed every hour. Mice were sacrificed at 0, 3, 6, 9, and 12 h after injection, 10 mice in each time point, and serum and liver tissue were collected.

### Overexpression and knockdown of *Cxcl1* in mice

The mice were randomly divided into 4 groups, 10 mice in each group, and the grouping conditions were as follows: Group 1: normal control group; Group 2: ACLF group; Group 3: *Cxcl1* overexpression group (CXCL1-OE); and Group 4: *Cxcl1* knockdown group (CXCL1-KD). Mice in group 1 were injected with olive oil twice a week for 8 weeks. Mice in groups 2, 3, and 4 were injected with CCl_4_/olive oil mixture twice a week for 4 weeks. To achieve *Cxcl1* overexpression and knockdown, 20 μg of *Cxcl1* overexpression adeno-associated virus (No. PGMAAV-11994, Genomeditech, Shanghai, China) and 20 μg *Cxcl1*-interfering adeno-associated virus (No. PGMAAV-10261, Genomeditech) were injected into the tail vein of mice in groups 3 and 4 in the 5th week. As controls, we set up a CXCL1-OE control group (n = 5) and a CXCL1-KD control group (n = 5). We infected ACLF mice with overexpression control AAV virus (No. PGMAAV-10320, Genomeditech, Shanghai, China) and interference control AAV virus (No. PGMAAV-11994, Genomeditech, Shanghai, China). The mice were then injected with the CCl_4_/olive oil mixture twice a week for another 4 weeks. At 24 hours after the last injection, mice were i.p. injected with a mixture of D-galactosamine (1000 mg/kg, Sigma) and LPS (100 ng/kg, Sigma), sacrificed 6 h later, and serum and liver tissue were collected.

### Statistics

GraphPad Prism 8 (GraphPad Inc., La Jolla, CA, USA) was used for graphing and data analysis. Two-tailed unpaired *t*-tests were used to compare differences between 2 groups, and 1-way ANOVA was used to compare differences among more than 2 groups. Statistical significance was set as follows: *p < 0.05, **p < 0.01, ***p < 0.001, ***p < 0.0001.

## RESULTS

### Characterization of the mouse model of ACLF

**FIGURE 1:**
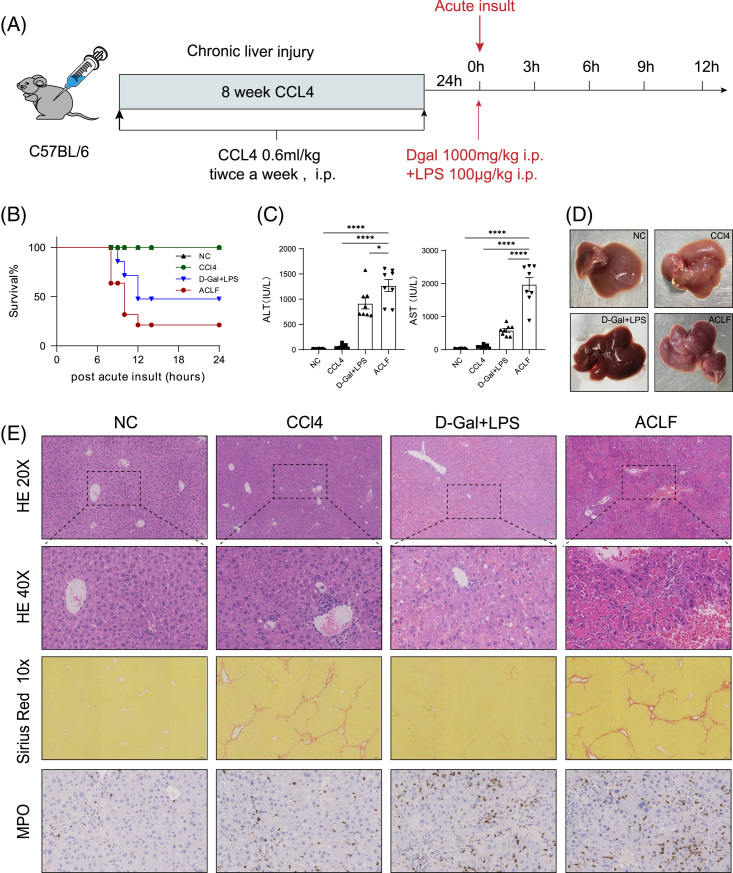
Characterization of the mouse model of ACLF. (A) Schematic schedule of ACLF mice model establishment. C57BL/6J mice were treated with CCl_4_ for 8 weeks to induce chronic liver injury, followed by an injection of D-gal/LPS. (B) Survival rates (n = 10). Group 1: group NC; Group 2: CCl4 group; Group 3: D-GalN + LPS; Group 4: ACLF group.ACLF group; i.p. with CCl_4_ for 8 weeks followed by i.p. with LPS and D-GalN once. (C)Serum ALT and AST levels at 6 hours after insult (n = 8). (D) Representative pictures of mouse livers in 4 groups. (E) H&E staining, Sirius Red staining, and MPO immunohistochemical staining of liver tissues in 4 groups. Representative images and summary data are shown. One-way ANOVA was used for statistical evaluation (*p < 0.05, **p < 0.01, ***p < 0.001, ****p < 0.0001). Abbreviations: ACLF, Acute-on-chronic liver failure; ALT, alanine aminotransferase; AST, aspartate aminotransferase; CCl4, carbon tetrachloride; D-GaIN, D-galactosamine; LPS, lipopolysaccharide; MPO, myeloperoxidase.

In this study, we used a CCl_4_ + LPS + D-GalN-induced liver injury mouse model to mimic the disease progression of ACLF (Figure 1A). Eight weeks of CCl_4_ injections mimicked the chronic fibrosis of the disease, and a single i.p. injection of LPS/D-GalN mimicked the course of i.p. bacterial infection and acute disease exacerbation. Mice were randomly divided into 4 groups, namely group NC, group CCl4, group D-GalN+LPS, and group ACLF, and the short-term mortality of the mice in the ACLF group reached 80% (Figure 1B). The serum alanine aminotransferase (ALT) and aspartate aminotransferase levels in the ACLF group were significantly increased compared with the other 3 groups (Figure 1C). The livers of mice in the ACLF group shrank, hardened in texture, and showed small nodules on the surface, accompanied by congestion and necrosis (Figure 1D). H&E staining showed extensive hepatocyte necrosis in the ACLF group, Sirius staining indicated liver fibrosis, and MPO immunohistochemistry showed massive neutrophil infiltration (Figure 1E). To investigate the changes in the systemic inflammatory response, we analyzed pro-inflammatory cytokines IL6, TNF-α, and IL-1β and anti-inflammatory cytokines IL-10, and the levels of serum cytokines in the ACLF group mice were significantly increased (Figure 4C, D). In addition, we found that terminal deoxynulceotidyl transferase nick-end-labeling staining in the ACLF group was strongly positive (Figure 5A), while the observation of JC-1-stained cells under a confocal microscope and flow cytometry showed that the mitochondrial membrane potential of hepatocytes in the ACLF group decreased (Supplemental Figure S1A, B, http://links.lww.com/HC9/A507), indicating that apoptosis may have occurred. The above data indicated that our ACLF mice had liver fibrosis, acute liver injury, cytokine storm, and poor prognosis, similar to the clinical manifestations of human ACLF.

### CXCL1 regulates neutrophil mobilization in ACLF

**FIGURE 2:**
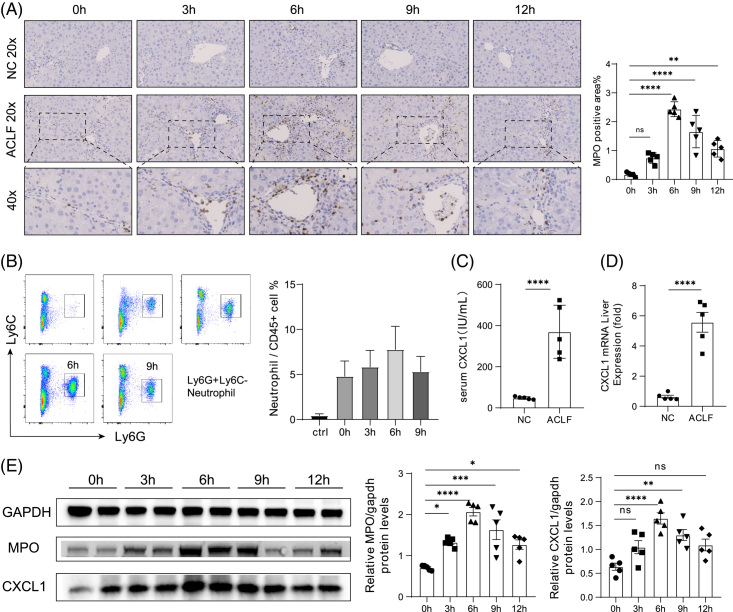
Liver inflammation is increased in the ACLF model mice. (A) MPO immunohistochemical staining of liver tissue of ACLF mice at different times after acute injury (n = 5). Representative images and summary data are shown. The Percentage of MPO + neutrophils was calculated by randomly counting 10 images (20×) per sample. (B) Ly6G + Ly6C- neutrophil counts in peripheral blood were analyzed using flow cytometry (n = 5). (C) The serum CXCL1 level was analyzed using ELISA (n = 5). (D) Hepatic *Cxcl1* expression was determined using quantitative real-time reverse transcription PCR (n = 5). (E) Hepatic levels of GAPDH, MPO, and CXCL1 were determined using immunoblotting analysis (left); and the blots were quantified by densitometry (right, n = 5). One-way ANOVA was used for statistical evaluation, *p < 0.05, **p < 0.01, ***p < 0.001, ***p < 0.0001. Abbreviations: ACLF, Acute-on-chronic liver failure; CXCL1, chemokine (C-X-C motif) ligand 1; D-GaIN, D-galactosamine; GAPDH, glyceraldehyde-3-phosphate dehydrogenase; MPO, myeloperoxidase; NC, normal control.

Neutrophils are the first response of the innate immune system and are rapidly recruited to the site of injury following acute injury. In addition to the CCl_4_+ Dgal/LPS-induced ACLF model reported here, we also detected a significant increase in CD45 ^+^ neutrophils in the APAP-induced ACLF mouse model (Supplemental Figure S2, http://links.lww.com/HC9/A507). And it has been reported that neutrophils also play a key role in ACLF caused by liver resection. To explore the underlying mechanism of neutrophil recruitment in ACLF, we analyzed MPO immunohistochemical staining at different time points after acute injury. MPO is a lysosomal protein present in the azurophilic granules of neutrophils and is an activation marker of neutrophils. We found that the amount of MPO + neutrophil infiltration gradually increased, reached a peak at 6 hours, and then decreased (Figure 2A). This result was also verified in flow cytometry analysis (Figure 2B), CD11b^+^Ly6G^+^ neutrophils in the peripheral blood of mice increased after acute injury and also reached a peak at 6 hours. We analyzed the expression of inflammatory mediators and chemokines that promote neutrophil infiltration in the serum of ACLF mice and found that the CXCL1 level was significantly increased (Figure 2C), and the mRNA expression of Cxcl1 in liver tissue was also enhanced (Figure 2D). We next performed a Western blotting analysis on liver tissue. Interestingly, we found a similar trend in the protein levels of CXCL1 and MPO, both peaking at around 6 hours and then decreasing (Figure 2E). Therefore, 6 hours were chosen as a critical time point in our ACLF model to investigate the underlying mechanism by which neutrophil recruitment by CXCL1 affects the progression of ACLF.

### Knockdown of CXCL1 reduces neutrophil recruitment and ROS generation, thereby attenuating hepatic inflammation and injury in ACLF

**FIGURE 3:**
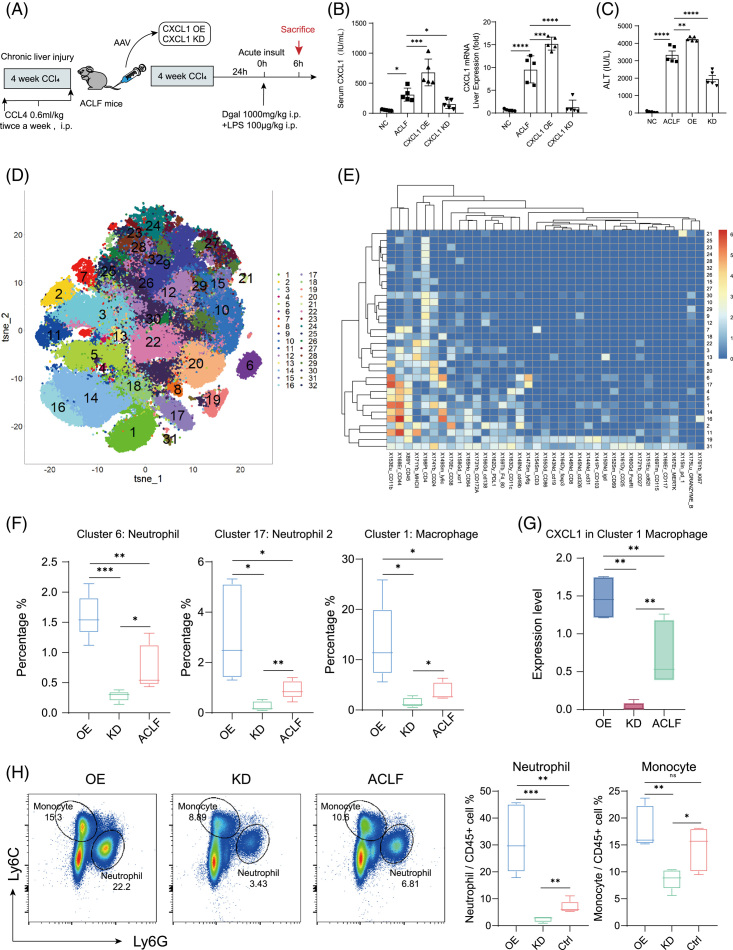
Cytometry by the time of flight (CyTOF) and flow cytometry reveal the local and systemic immune changes after AAV inoculation. (A) Schematic diagram of the injection of adenoviruses into the tail vein of ACLF mice. (B) Serum CXCL1 levels in each group were determined using ELISA (n = 5); The expression level of *Cxcl1* mRNA in the liver was determined using quantitative real-time reverse transcription PCR (n = 5). (C) Serum ALT in each group (n = 5). (D) Thirty-two major cell clusters are represented by a t-distributed stochastic neighbor embedding (t-SNE) plot based on CyTOF results. Each color was annotated to 1 major cell population. (E) Heatmap showing the average expression value of each marker in the 32 cell populations. (F) The percentages of neutrophils and macrophages differed among the 3 groups based on the CyTOF data (n = 5). The error bars represent the mean ± SEM; n.s., not significant, *p < 0.05 **p < 0.01 ***p < 0.005. (G) The level of CXCL1 produced by macrophages varied among the 3 groups based on the CyTOF data (n = 5). The error bars represent the mean ± SEM; n.s., not significant, *p < 0.05 **p < 0.01 ***p < 0.005. (H) The left 3 FACS plots show that the numbers of neutrophils and monocytes among mouse PBMCs varied among the groups. The 2 charts on the right show the corresponding statistical results (n = 5). The error bars represent the mean ± SEM; n.s., not significant, *p < 0.05 **p < 0.01 ***p < 0.005. Abbreviations: AAV, adeno associated virus; ACLF, Acute-on-chronic liver failure; ALT, alanine aminotransferase; CXCL1, chemokine (C-X-C motif) ligand 1; CyTOF, Cytometry by the time of flight; t-SNE, t-distributed stochastic neighbor embedding. Mass spectrometry antibody information is provided in Supplemental Material Table 2.

To explore the role of CXCL1 in neutrophil recruitment and host defense, ACLF mice were infected by tail vein injection of adeno associated virus (AAV) to achieve knockdown or overexpression of *Cxcl1* (Figure 3A). To ensure the infection efficiency of adeno-associated virus, 28 days after tail vein injection, we collected the livers of mice in the NC group, CXCL1 OE group, and CXCL1 KD group, sliced them and took pictures, and observed the expression of GFP in the livers (Supplemental Figure S3, http://links.lww.com/HC9/A507). Then, we analyzed the level of CXCL1 in the serum of mice in each group by ELISA and evaluated the relative mRNA expression level of CXCL1 in liver tissue by qRT-PCR. The results showed that the changes in the level of CXCL1 in the mouse liver and serum were in line with our expectations (Figure 3B). Interestingly, serum ALT also changed correspondingly according to CXCL1 levels, with serum ALT levels significantly decreased in the CXCL1-KD group and increased in the other group (Figure 3C). To exclude the influence of AAV vector itself, we infected ACLF mice with an empty virus and detected the expression level of CXCL1 mRNA in the liver of the CXCL1-OE control group and CXCL1-KD control group mice and their serum ALT, and found no difference with the ACLF group (Supplemental Figure S4A, B, http://links.lww.com/HC9/A507).

Then, mass cytometry and flow cytometry analysis revealed local and systemic immune changes in mouse liver after AAV inoculation. The 32 major cell clusters were represented by a t-distributed stochastic neighbor embedding plot on the basis of the CyTOF results (Figure 3D), in which each color represents a major cell population. The heatmap showed the average expression value of each marker in the 32 cell populations (Figure 3E). The percentages of neutrophils and macrophages differed among the 3 groups based on the CyTOF data (Figure 3F). The expression level of CXCL1 produced by macrophages also varied among the 3 groups based on the CyTOF data (Figure 3G). We found that the numbers of neutrophils and monocytes in the peripheral blood of the CXCL1-OE group were significantly increased compared with those in the CXCL1-KD group (Figure 3H).

**FIGURE 4:**
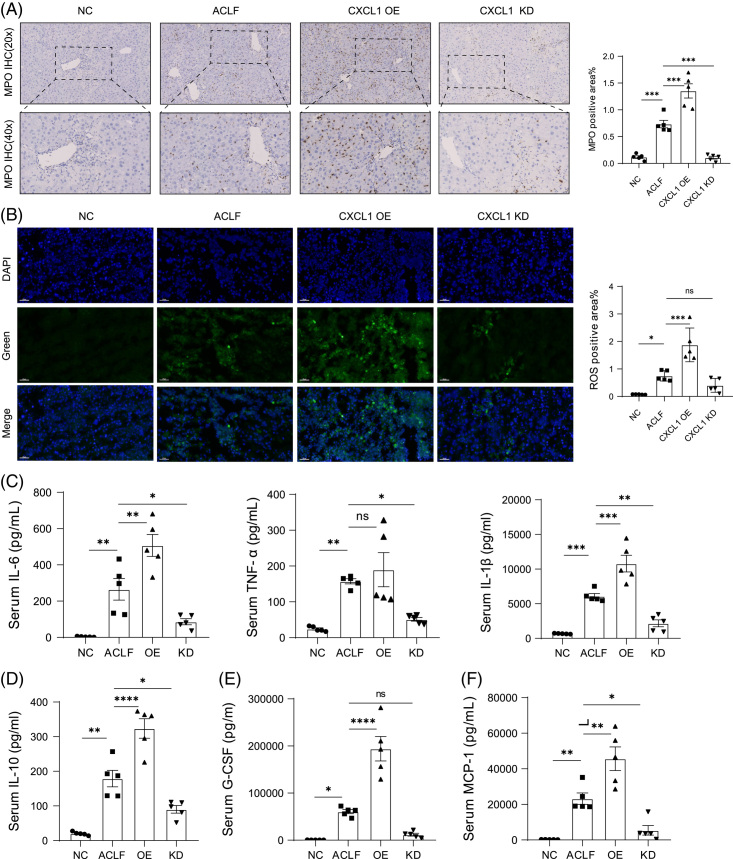
Hepatic overexpression of *Cxcl1* promotes ROS progression in ACLF mice. (A) MPO immunohistochemical staining of liver tissue (n = 5). Representative images and summary data are shown. The Percentage of MPO + neutrophils was calculated by randomly counting 10 images (20×) per sample. Scale bar, 50 μm. (B) Immunofluorescence staining of ROS in the NC, ACLF, CXCL1-OE, and CXCL1-KD groups (n = 5). Scale bar, 50 μm. (C–F) cytokines IL-6, TNF-α, IL-1β, IL-10, G-CSF, and MCP-1 were measured using ELISAs (n = 5). (*p < 0.05, **p < 0.01, ***p < 0.001, ***p < 0.0001). Abbreviations: ACLF, Acute-on-chronic liver failure; CXCL1, chemokine (C-X-C motif) ligand 1; G-CSF, granulocyte colony-stimulating factor; KD, knockdown; MCP-1, monocyte chemotactic protein 1; MPO, myeloperoxidase; OE, overexpression; ROS, reactive oxygen species.

Activated neutrophils can potently produce ROS. However, the overproduction of ROS can lead to oxidative breakdown, organelle damage, and even cell death. Studies have shown that ROS is largely involved in the pathological mechanism of liver disease. Our study showed that MPO+neutrophils were significantly upregulated in the CXCL1-OE group but downregulated in the CXCL1-KD group (Figure 4A). To determine whether ROS production was associated with CXCL1, we performed ROS immunofluorescence analysis on the liver tissues of each group. We found that there were a large number of ROS fluorescence signals diffusely in the liver of the CXCL1-OE group, and this phenomenon was significantly alleviated in the CXCL1-KD group (Figure 4B). To further investigate the changes in inflammatory responses, we analyzed the expression of pro-(IL6, TNF-α, IL-1β,and IFN-γ) and anti-(IL10) inflammatory cytokines in each group (Figure 4C, D and Supplemental Figure S4C, http://links.lww.com/HC9/A507). The results showed that after the combined treatment with CCL_4_+D-GalN+LPS, the levels of pro-inflammatory cytokines and anti-inflammatory cytokines in serum were significantly increased, and there was a correlation with the level of CXCL1. In addition, the levels of granulocyte colony-stimulating Factor (G-CSF) and monocyte chemotactic protein 1 were also significantly increased in the CXCL1-OE group and decreased in the CXCL1-KD group (Figure 4E, F). The above data indicate that the chemokine CXCL1 can effectively affect the recruitment of neutrophils and the generation of ROS. Knockdown of CXCL1 can reduce the recruitment of neutrophils, thereby reducing the generation of ROS, to alleviate liver inflammation and injury in ACLF.

### Knockdown of CXCL1 may alleviate liver injury in CCl4 + Dgal/LPS-induced ACLF mainly by attenuating hepatocyte apoptosis

**FIGURE 5:**
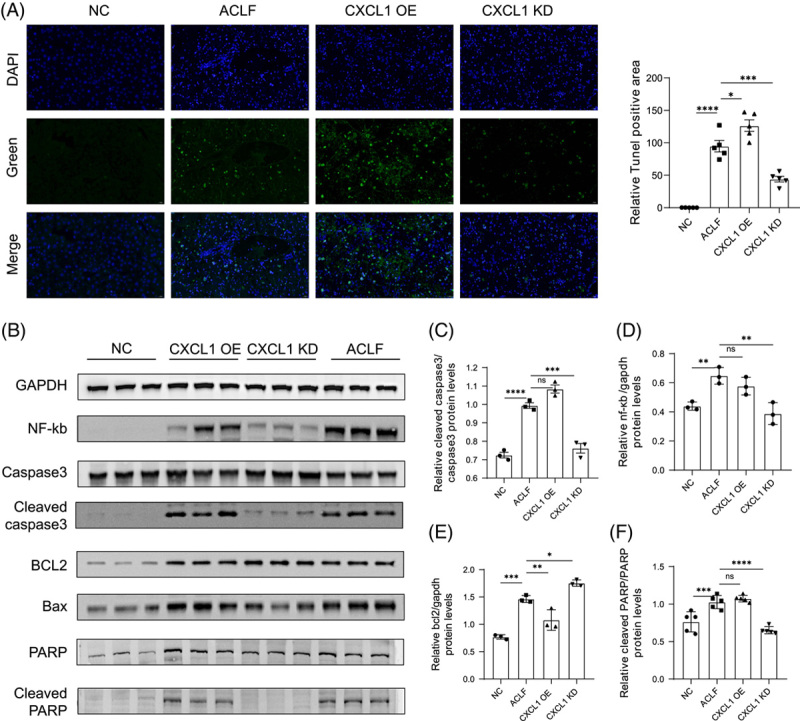
CXCL1 affects CCL4 + Dgal/LPS-induced liver injury in ACLF through the apoptosis signaling pathway. (A) Representative TUNEL staining in NC, ACLF, CXCL1-OE, and CXCL1-KD groups (n = 5). (B) Immunoblotting analysis of GAPDH, NF-κB, Caspase3, cleaved Caspase3, PARP, cleaved PARP, BAX, and BCL proteins in the liver. (C–F) Relative protein levels were measured using Image J. (*p < 0.05, **p < 0.01, ***p < 0.001, ***p < 0.0001). Abbreviations: ACLF, Acute-on-chronic liver failure; BAX, BCL2-associated X; BCL, B-cell lymphoma-2; CCl4, carbon tetrachloride; CXCL1, chemokine (C-X-C motif) ligand 1; KD, knockdown; MCP-1, monocyte chemotactic protein 1; LPS, lipopolysaccharide; OE, overexpression; PARP, poly ADP-ribose polymerase; ROS, reactive oxygen species; TUNEL, terminal deoxynulceotidyl transferase nick-end-labeling.

**FIGURE 6:**
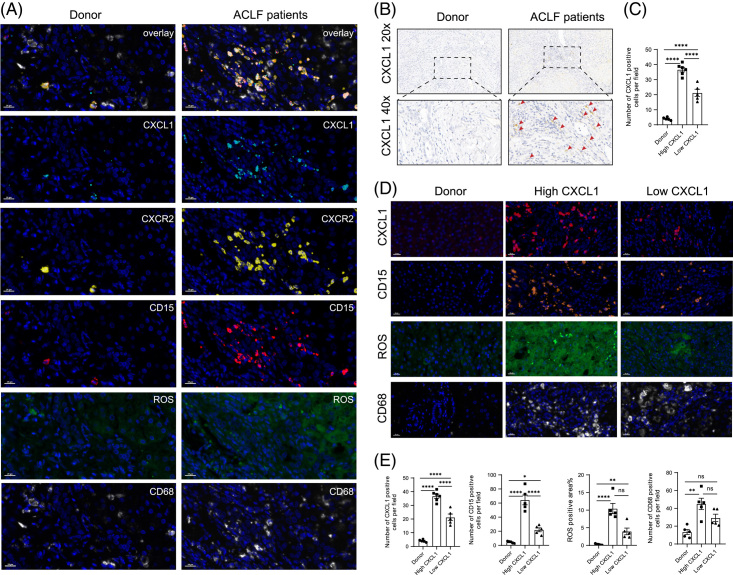
Multiplex immunohistochemistry revealed that CXCL1 is highly upregulated in the liver tissue of patients with ACLF and regulates ROS levels in the liver. (A) Representative images of multiplex immunostaining of livers from the healthy controls and patients with ACLF; the markers included CXCL1, CXCR2, ROS, CD15, and CD68 (n = 5). (B)Immunohistochemical staining for CXCL1 in the livers of the healthy controls and patients with ACLF. (C) Statistical analysis of CXCL1 immunohistochemical staining in the liver of the healthy control group and patients with ACLF (donor: n = 5; ACLF: n = 10), five 20× fields of view were selected for each sample. (D) Representative images of multiplex immunohistochemistry of the livers of the healthy controls, patients with high-CXCL1 ACLF, and patients with low-CXCL1 ACLF; the markers included CXCL1, ROS, CD15, and CD68. (E) Statistical analysis of CXCL1, ROS, CD15, CD68. Abbreviations: ACLF, Acute-on-chronic liver failure; CXCR2, chemokine receptor2; CXCL1, chemokine (C-X-C motif) ligand 1; ROS, reactive oxygen species.

The main cause of ACLF is a persistent inflammation of the liver and massive hepatocyte death. To explore the mechanism of ACLF liver injury caused by the massive accumulation of ROS in hepatocytes, we analyzed several main signaling pathways that may be involved, including apoptosis, necrosis, autophagy, etc. Terminal deoxynulceotidyl transferase nick-end-labeling staining indicated that the degree of apoptosis in the liver of ACLF mice was significantly increased, and knockdown of *Cxcl1* could significantly reduce the apoptosis induced by CCl_4_+D-GalN+LPS, whereas overexpression of *Cxcl1* promoted hepatocyte apoptosis (Figure 5A). Neutrophils produce large amounts of ROS during phagocytosis of pathogens in both mice and human livers (Figures 4B, 6D), which can cause DNA damage and possibly induce apoptosis. For further verification, we detected the levels of several apoptotic pathway key proteins in the liver tissues of mice in the NC group, ACLF group, CXCL1-OE group, and CXCL1-KD group, including NF-κB, caspase3, cleaved caspase3, BCL2, BAX, PARP, and cleaved PARP (Figure 5B). Western blotting analysis indicated that the protein levels of cleaved caspase3, BAX, and cleaved PARP were significantly increased in ACLF mice versus control mice, while the expression level of BCL-2 decreased, indicating that hepatocyte apoptosis is activated (Figure 5C–F). Knockdown of *Cxcl1* reduced the protein levels of NF-κB, cleaved caspase3, cleaved PARP, and BAX and alleviated apoptosis of hepatocytes. We then detected key regulators of necrosis: receptor-interacting protein kinase 3 (RIPK3) and mixed lineage kinase domain. It was found that the levels of RIPK3 and mixed lineage kinase domain were indeed significantly upregulated in the ACLF group compared with the NC group but not related to the knockdown and overexpression of CXCL1. Moreover, phosphorylated RIPK3 and phosphorylated mixed lineage kinase domain were barely detectable, suggesting that this pathway was not activated (Supplemental Figure S5, http://links.lww.com/HC9/A507). The autophagy-related marker protein LC3BII/I was also increased in the ACLF group, but there was no significant difference in the CXCL1-OE group and CXCL1-KD (Supplemental Figure S5, http://links.lww.com/HC9/A507). These results demonstrate that knockdown of CXCL1 may alleviate liver injury in ACLF mainly by attenuating hepatocyte apoptosis, rather than through pathways such as cell necrosis and autophagy.

### Multiplex immunohistochemistry revealed that CXCL1 is highly upregulated in the liver tissue of patients with ACLF and regulates the ROS levels in the liver

In our previously published study, we showed that serum CXCL1 levels and neutrophil counts were significantly higher in patients with HBV-ACLF than in healthy individuals and patients with chronic HBV or HBV-ACLF–compensated cirrhosis. However, it has not been validated in liver tissue from patients with ACLF. We collected liver tissues from 5 healthy controls and 10 patients with ACLF for further validation. Immunohistochemical staining for CXCL1 was performed. We found that CXCL1 was hardly expressed in the livers of the healthy controls, whereas it was highly upregulated in the livers of patients with ACLF (Figure 6B). However, conventional immunohistochemistry can only show 1 marker in tissue sections. To explore the interaction between cells more intuitively, we performed mIHC using the markers CXCL1 (cyan), CXCR2 (yellow), CD15 (red), ROS (green), CD68 (white), and DAPI (nuclei, blue) (Figure 6A). Neutrophils were labeled with CD15 and macrophages were labeled with CD68. The results indicate that CXCL1 levels were significantly elevated in the liver tissue of patients with ACLF. In addition, we found that the location of CXCR2 staining almost completely coincided with the location of CD15 staining, indicating that CXCR2 was expressed on the surface of CD15 + neutrophils. The positions of CXCL1 and CXCR2/CD15 also partially matched, indicating that CXCL1 recruits neutrophils by binding to CXCR2 on their surface. Subsequently, we divided the patients with ACLF into 2 groups according to their expression level of CXCL1, among which the higher 5 were High-CXCL1, and the rest were Low-CXCL1 group (Figure 6C, D), and found that the neutrophil level and ROS level in the High-CXCL1 group were significantly higher than those in the Low-CXCL1 group. There was no significant difference in the number of macrophages between the groups (Figure 6E). In short, higher levels of CXCL1 recruit more neutrophils by binding to CXCR2, producing a mass of ROS to destroy liver cells.

## DISCUSSION

**FIGURE 7:**
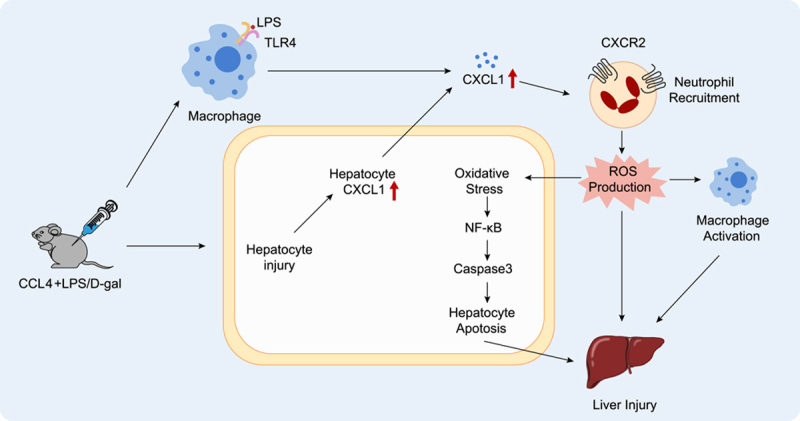
Schematic diagram of CXCL1 recruitment of CXCR2 + neutrophils to the liver, which increases ROS levels and aggravates caspase3-induced hepatocyte apoptosis, thereby exacerbating liver injury. Abbreviations: CXCR2, chemokine receptor2; CXCL1, chemokine (C-X-C motif) ligand 1; ROS, reactive oxygen species.

An excessive systemic inflammatory response is an important feature of ACLF, in which massive infiltration of neutrophils producing abundant ROS, and the necrosis and apoptosis of hepatocytes are the important mechanisms leading to poor prognosis.^22,23^ Our findings confirmed that the level of CXCL1 was significantly upregulated in both patients with ACLF and ACLF mice, recruiting more neutrophils to produce ROS, which led to the death of hepatocytes. In ACLF mice, overexpression of *Cxcl1* markedly increased the number of hepatic neutrophils and aggravated oxidative stress, which in turn activated the downstream caspase3 pathway, leading to hepatocyte apoptosis, liver injury, and inflammation. Knockdown of *Cxcl1* reduced the degree of neutrophil infiltration, ROS production, and hepatocyte apoptosis in liver tissue. Therefore, CXCL1 might be a key factor affecting the prognosis of ACLF. Here, we constructed a schematic diagram of the proposed CXCL1-mediated hepatic inflammation and injury in the hepatic microenvironment of ACLF (Figure 7).

Our animal model combines a double-hit regimen based on long-term CCl_4_ treatment and injections of D-GalN and LPS to mimic chronic liver fibrosis and bacterial infection and acute liver injury in the course of ACLF. Carbon tetrachloride is a hepatotoxic drug and is typically used to cause liver damage in experimental animals.^24^ LPS is a constituent of the outer cell wall of gram-negative bacteria. In infections caused by gram-negative bacteria (such as *Escherichia coli* and *Salmonella typhimurium*), LPS stimulates the release of inflammatory factors by binding to toll-like receptor 4 on the surface of macrophages.^25^ Compared with certain existing models, the ACLF mouse model in this study includes features such as chronic liver injury, acute liver injury, bacterial infection, massive neutrophil infiltration, cytokine storm, hepatocyte damage, necrosis and apoptosis, and poor prognosis, which mimics the complex prognostic situation of clinical ACLF.

Neutrophils act as the first line of defense at sites of infection and tissue damage, and extensive neutrophil infiltration is a feature of ACLF. Interestingly, Stefanovic’s study revealed that in a CCl_4_-induced liver injury model, the chemokine CXCL1 did not induce hepatic neutrophil infiltration.^18^ However, in this study, we provided evidence that CXCL1 plays a key role in neutrophil recruitment and, by promoting hepatic neutrophil infiltration, contributes to liver injury. First, in the CCl_4_+D-GalN+LPS-induced ACLF mouse model, the level of CXCL1 was observably up-regulated in serum and liver tissue, and the results of immunohistochemical staining and flow cytometry confirmed that neutrophils were also significantly elevated. Then, in the ACLF mouse model, we overexpressed and knocked down the *Cxcl1* gene in the mouse liver using AAV. In the CXCL1-OE group, the degree of hepatic neutrophil infiltration was markedly increased, whereas, in the CXCL1-KD group, it was decreased. Furthermore, we found that the level of G-CSF was also significantly increased in the CXCL1-OE group. G-CSF mainly acts on the proliferation, differentiation, and activation of the neutrophil lineage and is one of the most important growth factors for the production of granulocytes and their immediate precursors.^26^ Our study confirmed that the level of G-CSF in the serum of mice was significantly increased in the case of overexpression of *Cxcl1*, while the level of G-CSF was markedly decreased in the *Cxcl1* knockdown group. Correspondingly, flow cytometry showed that the number of neutrophils in the peripheral blood of the CXCL1-OE group was significantly higher than that of the CXCL1-KD group. At the same time, we performed multiplex immunohistochemistry in the liver samples of patients with ACLF to verify this finding, which showed that both CXCL1 levels and neutrophil levels were significantly increased in patients with ACLF. Our findings also provided visual confirmation that CXCL1 binds to CXCR2 on the surface of CD15+ neutrophils to stimulate neutrophil recruitment. A study demonstrated that blocking CXCR2 inhibited hepatic neutrophil infiltration and injury in a chronic ethanol feeding model precisely because CXCR2 is a receptor for CXCL1.^27^ In addition, when patients with ACLF were grouped by their CXCL1 levels, their liver ROS levels also correlated positively with CXCL1 levels.

Our results demonstrate that in ACLF mice, the mitochondrial membrane potential of hepatocytes is depolarized, and there is a large amount of ROS diffusely present in the liver. Further, we examined and analyzed liver tissue from patients with ACLF and found a significant increase in ROS. In ACLF mice, the level of ROS in the CXCL1-OE group was significantly upregulated; and the level of ROS in the CXCL1-KD group was significantly downregulated. After being recruited, neutrophils recognize, bind, and phagocytose pathogens through pathogenic pathogen-associated molecular patterns. During phagocytosis, the NADPH oxidase complex is activated and releases antimicrobial peptides, proteases, and a large amount of ROS to kill and destroy bacteria.^28^ ROS are a normal by-product of aerobic metabolism and play a vital decision in some pathological processes, and excessive ROS can cause oxidative damage to cells, inhibit cellular function, induce mitochondrial phagocytosis, and induce apoptosis.^29^ The liberation of cellular contents further exacerbates inflammatory injury.^30^ In addition, ROS promotes the secretion of cytokines, which in turn leads to an increase in the production of ROS, forming a vicious circle and further promoting liver damage. The massive accumulation of neutrophils and excessive production of ROS have been reported to be one of the important causes of various inflammation-related liver diseases, such as alcohol-associated liver disease, hepatic ischemia/reperfusion injury, HCC, and ACLF.^8,19,31,32^ In addition, damage-associated molecular patterns released after ROS lead to necrosis and apoptosis of hepatocytes. These damage-associated molecular patterns activate innate immune cells in the liver through pattern recognition receptors to produce inflammatory cytokines, including IL-6, TNF-α, and IL-1β, and amplify the inflammatory response, thereby aggravating ACLF. To investigate whether knockdown of *Cxcl1* is associated with the inhibition of pro-inflammatory cytokines in ACLF, we detected IL-6, TNF-α and IL-1β in the serum of each group. As expected, in the *Cxcl1* knockdown group, they were markedly reduced, which alleviated the hepatic inflammatory response.

The next question is, “By which pathway does ROS affect the severity and prognosis of ACLF?” Studies have indicated that ROS-induced lipid peroxidation plays a key role in cell death and participates in the regulation of apoptosis, autophagy, and ferroptosis.^33^ After detecting and analyzing certain marker molecules of each pathway, we found that under the influence of different levels of CXCL1, the level of hepatocyte apoptosis in ACLF mice changed significantly. The results of terminal deoxynulceotidyl transferase nick-end-labeling staining showed that there was abundant hepatocyte apoptosis in the liver tissue of ACLF mice, and when *Cxcl1* was overexpressed, the degree of apoptosis was further aggravated, and when Cxcl*1* was knocked down, the level of liver apoptosis was weakened. This result indicated that antagonizing chemokine CXCL1 would be beneficial to ameliorate the degree of apoptosis of hepatocytes caused by excessive ROS production. Further Western blotting analysis showed that the protein levels of NF-κB, cleaved caspase3, cleaved PARP, and BAX decreased in the CXCL1-KD group, indicating that antagonizing CXCL1 has a marked anti-apoptotic effect. PARP is closely related to DNA repair, and its cleavage is considered to be an important indicator of apoptosis. Activated caspase-3 cleaves PARP, thereby promoting apoptosis. BAX is a pro-apoptotic protein whose overexpression can antagonize the protective effect of BCL-2, increasing the tendency of cells to die. It has been demonstrated that lipid peroxidation products can induce apoptosis through different signaling pathways, including the NF-κB pathway, MAPK pathway, and protein kinase C pathway. The NF-κB protein family is widely involved in inflammation, stress response, survival, and cell death and is also responsible for the transcriptional regulation of anti-apoptotic gene expression.^34^ We also analyzed other cell death pathways. The key regulators of cell necrosis RIPK3 and RIPK3 were indeed up-regulated in the ACLF group, but they were not correlated with the level of CXCL1, and their phosphorylated active proteins were not detected. Autophagy-related marker protein LC3BII/I also did not change significantly. Consequently, we propose that intervention for CXCL1 could affect the prognosis of ACLF by decreasing neutrophil recruitment and infiltration to reduce ROS production, thereby regulating NF-κB and alleviating the downstream caspase3 apoptotic pathway, rather than through pathways such as cell necrosis and autophagy.

In summary, we found that CXCL1 plays an important role in ACLF. First, the expression level of CXCL1 was significantly upregulated in both ACLF mice and patients with ACLF. Second, multiplex immunohistochemistry confirmed that CXCL1 recruits neutrophils by binding to CXCR2 on their surface. Third, the knockdown of *Cxcl1* ameliorated CCL4+D-GalN+LPS-induced hepatic neutrophil infiltration and injury, improved ROS levels, and reduced hepatocyte apoptosis.

In summary, we successfully constructed a model of ACLF and demonstrated that the expression level of CXCL1 was significantly upregulated in both ACLF mice and ACLF patients. The chemokine CXCL1 regulates neutrophil mobilization by binding to CXCR2 on the neutrophil surface, generating massive oxidative stress, mitophagy, and induction of caspase-associated hepatocyte apoptosis. Whereas Cxcl1 knockdown improved neutrophil infiltration, alleviated ROS levels, and reduced hepatocyte apoptosis, thereby attenuating inflammation and liver injury in ACLF. These results provide a theoretical basis for ACLF research and may provide new ideas for the clinical treatment of ACLF.

## Supplementary Material
