# Supplementary material for: Knockdown of CXCL1 improves ACLF by reducing neutrophil recruitment to attenuate ROS production and hepatocyte apoptosis
Source: Hepatol Commun. 2023 Sep 15;7(10):e0257. doi: 10.1097/HC9.0000000000000257 (PMC10503672; doi:10.1097/HC9.0000000000000257)
Supplement: Supplementary file 1 [file hc9-7-e0257-s001.pdf]

Supporting Materials

Knockdown of CXCL1 Improves ACLF by Reducing Neutrophil Recruitment to Attenuate ROS Production and Hepatocyte Apoptosis

Shima Tang[1]*, Junlei Zhang[2]*, Lingjian Zhang[3]*, Yalei Zhao[4], Xiao Lanlan[1], Fen Zhang[1], Qian Li[1], Ya Yang[1], Qiuhong Liu[1], Lanjuan Li[1#]

Affiliations:

1. State Key Laboratory for Diagnosis and Treatment of Infectious Diseases, National Clinical Research Center for Infectious Diseases, Collaborative Innovation Center for Diagnosis and Treatment of Infectious Diseases, The First Affiliated Hospital, Zhejiang University School of Medicine, 310003, Hangzhou, China

2. Zhejiang Provincial Key Laboratory of Pancreatic Disease, the First Affiliated Hospital, Zhejiang University School of Medicine, 310002, Hangzhou, China

3. Department of Infectious Diseases, The Affiliated Hangzhou First People's Hospital, School of Medicine, 310002, Zhejiang University, Hangzhou, China

4. Department of Infectious Diseases, The First Affiliated Hospital of Xi'an Jiaotong University, Xi'an, China

Supplemental Methods:

Model of APAP-induced ACLF

The mice were randomly divided into two groups: NC group and APAP-ACLF group. The NC group (n=6) were continuously injected with olive oil for 8 weeks, and sacrificed after the last injection, and the serum and liver tissue were collected. In the APAP-ACLF group (n=6), mice were intraperitoneally injected with a mixture of CCl_4 (0.6ml/kg, Aladdin, Shanghai, China) and olive oil for 8 weeks. Twenty-four hours after the last injection, APAP (600 mg/kg, lot111169, MCE, USA) was injected intraperitoneally, sacrificed 6 hours later, and serum and liver tissue were collected.

Liver injury analysis

Serum alanine aminotransferase (ALT) and aspartate aminotransferase (AST) were analyzed using a kit from the Nanjing Jiancheng Bioengineering Institute (Nanjing, China) following the

suppliers' the operating instructions. Mouse liver tissue was collected at each 0, 3, 6, 9, and 12 h after acute injury, fixed and embedded in paraffin. Sections of the paraffin blocks were placed on glass slides and stained with hematoxylin and eosin (H&E).

Mouse multiplex cytokine and chemokine analysis

Mouse serum cytokines were measured using the Bio-Plex Pro Mouse Cytokine 23-Plex Immunoassay (Bio-Rad, Hercules, CA, USA) according to the standard protocol, including CXCL1, interleukin (IL)-6, IL-1b, tumor necrosis factor alpha (TNF-α), IL-10 and Interferon- γ (IFN γ).

Quantitative real-time reverse transcription PCR (qRT-PCR)

Total RNA from liver tissue was extracted using Trizol (15596026, Thermo Fisher Scientific, Waltham, MA, USA) following standard procedures according to the manufacturer's instructions. qRT-PCR was performed on a real-time PCR system (Applied Biosystems, Foster City, CA, USA) using the One Step PrimeScript™ RT-PCR Kit (Takara, Shiga, Japan). Real-time PCR was performed using the SYBR Premix Ex Taq II kit (Takara, Shiga, Japan). Three replicate wells were set for each sample, and then the mean was calculated to normalize relative mRNA expression to that of *Gapdh* (encoding glyceraldehyde-3-phosphate dehydrogenase). The primer sequences are listed in Supplementary Table 1.

Western blotting analysis

Liver tissue was triturated and lysed in Radioimmunoprecipitation assay (RIPA) lysis buffer (Beyotime Biotechnology, Shanghai, China) containing 1% protease and a phosphatase inhibitor cocktail to obtain the proteins. About 20 μg of protein sample was separated on 4–20% precast mini- polyacrylamide gels (Genscript, Nanjing, China) using sodium dodecyl sulfate-polyacrylamide gel electrophoresis (SDS-PAGE) and then transferred to Immun-Blot polyvinylidene fluoride (PVDF) membranes (Bio-Rad). The primary antibodies used were: anti-myeloperoxidase (MPO; ab208670; 1:1000, Abcam, Cambridge, MA, USA), anti-CXCL1 (BAF453;1:2000, R&D Systems, Inc., Minneapolis, MN, USA), anti-GAPDH (5174), anti-poly(ADP-ribose) polymerase (PARP) (9532S), anti-cleaved PARP(94885), anti-nuclear factor kappa B (NF-κB) (8242T), anti-caspase3(9662S), anti-cleaved caspase3 (9509T), anti-B-Cell CLL/lymphoma 2 (BCL2) (3498S), anti-BCL2 associated X protein (BAX) (14796), anti-caspase 8 (9508S), anti-Mixed lineage kinase domain-like protein (MLKL, 37705),

anti-ph-MLKL (38333), anti-Receptor-interacting protein 3 (RIP3, 15828), anti-ph-RIP3 (9170s) (1:1000, Cell Signaling Technology, USA), and anti-Microtubule-Associated Protein 1 Light Chain 3 (LC3, 14600-1-AP, proteintech). Then incubated with anti-mouse HRP-linked antibody (7074S) and Anti-rabbit HRP-linked antibody (7076S, Cell Signaling Technology, USA). Then incubated with anti-mouse HRP-linked antibody(7074S) and Anti-rabbit HRP-linked antibody (7076S, Cell Signaling Technology, USA). The immunoreactive proteins were detected using an enhanced chemiluminescence (ECL) kit (Beyotime Biotechnology) and analyzed using ImageJ (NIH, Bethesda, MD, USA).

Terminal deoxynulceotidyl transferase nick-end-labeling (TUNEL) staining

TUNEL assays (Vazyme, Nanjing, China) were used to assess apoptosis of liver tissues according to the manufacturer's instructions. Briefly, liver paraffin sections were deparaffinized and rehydrated, then 20 μg/mL protease K solution was added at room temperature and incubated for 20 min. The sections were washed thrice using phosphate-buffered saline (PBS), and then immersed in equilibration buffer for 30 min before incubating with TUNEL reaction mixture in a humidified chamber at 37 °C for 1 h. The reactions were stopped, then the sections were stained using 4′,6-diamidino-2-phenylindole (DAPI) at room temperature for 5 min. A PANNORAMIC 250 Flash series digital scanner (3DHISTECH, Budapest, Hungary) was used to scan the slides. Five visual fields (40 × magnification) were randomly selected on each slide, and ImageJ software was used to determine the index of the apoptosis cell rate.

Fluorescence activated cell sorting (FACS) analysis

Tissues were digested for 1 h at 37 °C in medium containing 0.01 mg/mL DNase I (11284932001, Merck, Rahway, NJ, USA) and 0.6 mg/mL collagenase IV (17104019, Gibco, Grand Island, NY, USA). Digested tissue was filtered through a 0.4 μm cell strainer (352340, BD Biosciences, San Jose, CA, USA) to obtain a single-cell suspension. Then, the cells were centrifuged at 300 × *g* for 5 min and resuspended in 36% Percoll (P4937, Sigma). After another centrifugation, cells were treated in lysis buffer (555899, BD Bioscience) to separate red blood cells. Then, the cells were preincubated with Fc blocker (156604 for mice, Biolegend, San Diego, CA, USA) for 10 minutes. Thereafter, the suspension was incubated with fluorochrome-labeled antibodies (Table S2) for 30 min at 4 °C. The cell suspensions were analyzed on a Pentalaser flow cytometer (BD Biosciences). Data were analyzed using FlowJo software (TreeStar, Ashland. OR, USA).

ROS detection

The CellROX™ Kit (C10492, Thermo Fisher Scientific) was used to detect ROS in tissue sections on slides. The ROS probe was incubated with the slides for 30 min before DAPI staining. Then, the slides were visualized using the Vectra Polaris Quantitative Pathology Imaging Systems (Akoya, Boston, MA, USA).

Measurement of mitochondrial membrane potential.

Changes in MMPs were measured using the fluorescent dye 5',6,6'-tetrachloro-1,1',3,3'-tetraethyl-imidacarbocyanine iodide (JC-1 fluorescent dye, Beyotime, China). First, hepatocytes were isolated from livers. For IF imaging, pre-attach hepatocytes to glass slides for 24h. After cell treatment, 5 μM JC-1 stain was added to the cells and incubated for 30 min at 37°C in the dark. Cells labeled with JC-1 were visualized with a confocal fluorescence microscope (Leica TCS SP8X, Germany) and examined at 530 nm (JC-1 monomeric form, green) and 590 nm (JC-1 aggregated form, red). For flow-cytometry detection, 5 μM JC-1 stain was added to the cells and incubated for 30 min at 37°C in the dark. Isolated hepatocytes were detected by a five-laser flow cytometer (BD Biosciences Fortessa, USA).

Multiplex immunohistochemistry (mIHC)

We used the Opal Polaris ™ T 7-color Manual IHC Kit (NEL861001KT, Akoya). The main antibodies used for mIHC included those recognizing CXCR2 (ab225732, Abcam), CXCL1 (ab89318, Abcam), CD68 (ab213363, Abcam), and CD15 (376302, Biolegend). After DAPI staining, visualization of the slides was performed by Vectra Polaris Quantitative Pathology Imaging Systems (Akoya).

Cytometry by time of flight (CyTOF)

Tissue was digested in culture medium supplemented with 0.6 mg/mL collagenase IV (17104019, Gibco) and 0.01 mg/mL DNase I (11284932001, Merck) at 37 °C for 1 h. Digested tissue was filtered through a 0.4 μm cell strainer (352340, BD Biosciences), followed by centrifugation at 300 × *g* for 5 min and resuspension in 36% Percoll (P4937, Sigma). After another round of centrifugation, the cells were resuspended in 10 mL of blood lysis buffer (555899, BD Biosciences) for 10 min at room temperature to remove red blood cells. The cell suspension was centrifuged again and preincubated with 2.5 μg/mL Fc blocker (156604, Biolegend) for 10 minutes on ice. Then, the suspensions were further mixed with extracellular metal-labelled

antibodies at room temperature for 30 min. The antibodies are listed in Table S1. A Forkhead box P3 (FOXP3)/Transcription Factor Staining Buffer Set (00-5523-00, Thermo Fisher Scientific) was utilized before intracellular antibody incubation. After all antibody staining, nuclear marking was performed using 125 nM Ir (201192B, Fluidigm, South San Francisco, CA, USA) at 4 °C overnight. Then, two washes with Cell Staining Buffer and one with ddH2O were performed. The cell number was adjusted to 1.1×10^6/mL using Cell Acquisition Solution (201240, Fluidigm) and 10% EQ beads (201245, Fluidigm) were added into the solution. Finally, the suspensions were collected and analyzed using a CyTOF machine (Helios, Fluidigm).

CyTOF data analysis

CyTOF data were pre-processed using FlowJo to manually gate the CD45+ cell population from Flow cytometry standard (FCS) files. Major cluster re-clustering was performed using the cytofkit package in the R software. PhenoGraph, using the default parameters, was applied for sub-cluster identification. Visualization was implemented in ggplot2 in the R software.

Supplemental Table 1:

GAPDH: AGATCCCTCCAAAATCAAGTGG GGCAGAGATGATGACCCTTTT

CXCL1: TCTCCGTTACTTGGGGAC CCACACTCAAGAATGGTCGC

Supplemental Table 2：Mass Spectrometry Antibody Information Sheet

**CyTOF Antibodies**						
**Antibodies**	**Clone**	**Source**	**Cat#**	**Metals**	**Source**	**Cat#**
CD45	30-F11	Biolegend	103141	89Y	Macklin	Y820645
PD-1	RMP1-30	Biolegend	109113	115ln	Macklin	I811750
CXCR2	SA044G4	Biolegend	149302	139La	Macklin	L812522
CD103	2E7	Biolegend	121402	141 Pr	Fluidigm	201141A
CXCL1	1174A	R&D	MAB4532-100	142 Nd	Fluidigm	201142A
CD19	6D5	Biolegend	115547	143 Nd	Fluidigm	201143A
CD31	390	Biolegend	102425	144 Nd	Fluidigm	201144A
CD326	G8.8	Biolegend	118223	145 Nd	Fluidigm	201145A
CD8	53-6.7	Biolegend	100755	146 Nd	Fluidigm	201146A

Ly6G	1A8	Biolegend	127637	147 Sm	Fluidigm	201147A
CD49b	HMα2	Biolegend	103513	148 Nd	Fluidigm	201148A
Ly6C	HK1.4	Biolegend	128039	149 Sm	Fluidigm	201149A
IgD	11-26c.2a	Biolegend	405737	150 Nd	Fluidigm	201150A
CD62L	MEL-14	Biolegend	104443	151 Eu	Fluidigm	201151A
CD69	H1.2F3	Biolegend	104533	152 Sm	Fluidigm	201152A
CD11b	M1/70	Biolegend	101249	153 Eu	Fluidigm	201153A
CD3	145-2C11	Biolegend	100345	154 Sm	Fluidigm	201154A
CD86	GL-1	Biolegend	105002	155 Gd	Fluidigm	201155A
CD138	281-2	Biolegend	142502	156 Gd	Fluidigm	201156A
XCR1	ZET	Biolegend	148202	158 Gd	Fluidigm	201158A
F4/80	BM8	Biolegend	123143	159 Tb	Fluidigm	201159A
FceRI	MAR-1	Biolegend	134321	160 Gd	Fluidigm	201160A
CD25	3C7	Biolegend	101913	161 Dy	Fluidigm	201161A
PD-L1	10F.9G2	Biolegend	124302	162 Dy	Fluidigm	201162A
CD11c	N418	Biolegend	117341	163 Dy	Fluidigm	201163A
Foxp3	MF-14	Biolegend	126402	164 Dy	Fluidigm	201164A
CD64	W18349C	Biolegend	164402	165 Ho	Fluidigm	201165A
CD117	2B8	Biolegend	105829	166 Er	Fluidigm	201166A
Mertk	2B10C42	Biolegend	151502	167 Er	Fluidigm	201167A
CD44	IM7	Biolegend	103051	168 Er	Fluidigm	201168A
CD115	AFS98	Biolegend	135521	169 Tm	Fluidigm	201169A
CD38	90	Biolegend	102723	170 Er	Fluidigm	201170A
MHC II	M5/114.15.2	Biolegend	107637	171 Yb	Fluidigm	201171A
CD27	LG.3A10	Biolegend	124202	172 Yb	Fluidigm	201172A
CD172a	P84	Biolegend	144002	173 Yb	Fluidigm	201173A
CD24	M1/69	Biolegend	101829	174 Yb	Fluidigm	201174A
Granzyme B	QA16A02	Biolegend	372202	175 Lu	Fluidigm	201175A
Ki-67	11F6	Biolegend	151202	176 Yb	Fluidigm	201176A

CD4	RM4-5	Biolegend	100561	198 Pt	Fluidigm	201198

Supplemental Figure 1:

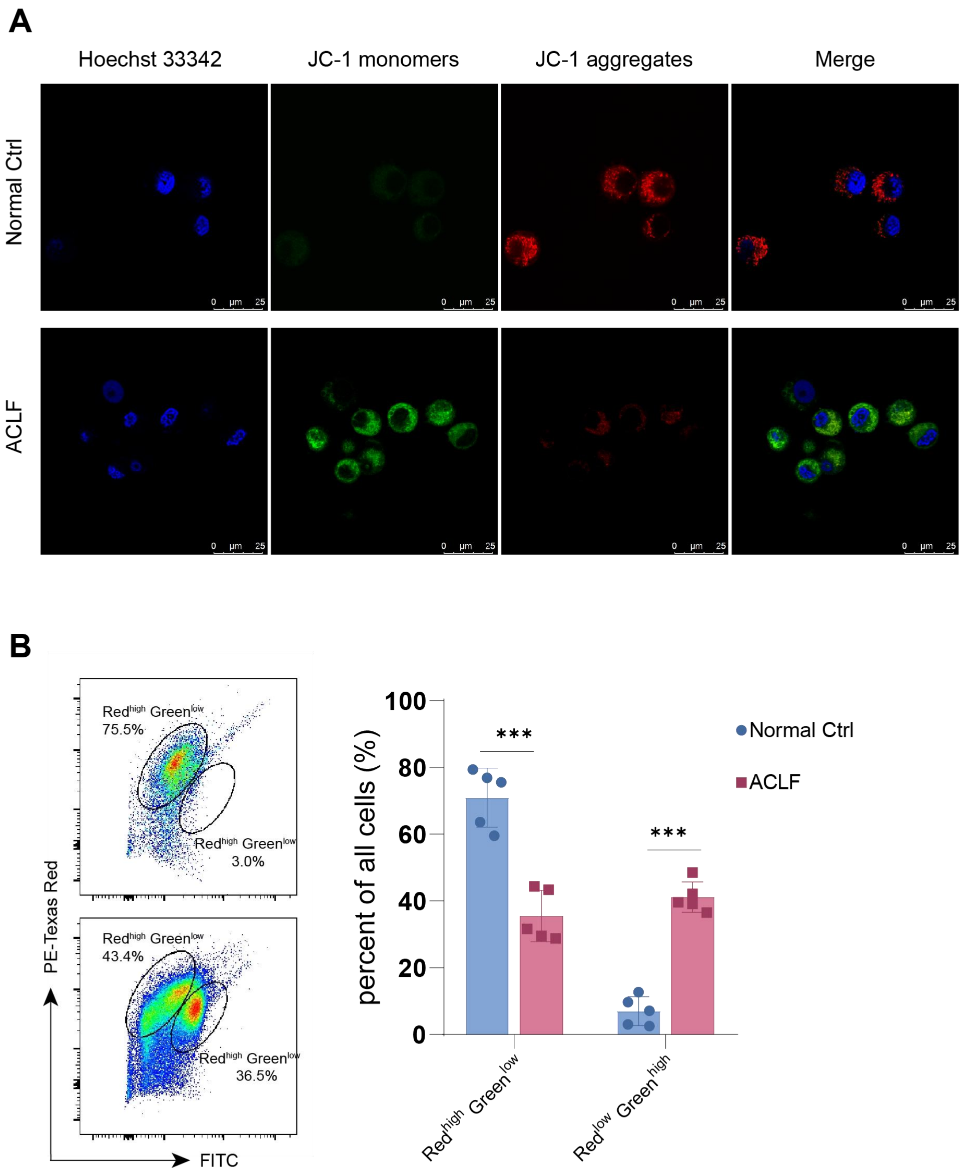

Figure S1. (A) The mitochondrial membrane potential of hepatocytes in the NC group and the ACLF group was detected by JC-1 staining. Representative confocal micrographs are shown. Bar = 25 μm. (B)JC-1 was used to detect the mitochondrial membrane potential of hepatocytes in NC group and ACLF group by flow cytometry (n=5). *P < 0.05, **P < 0.01, ***P < 0.001, ***P < 0.0001.

Supplemental Figure 2:

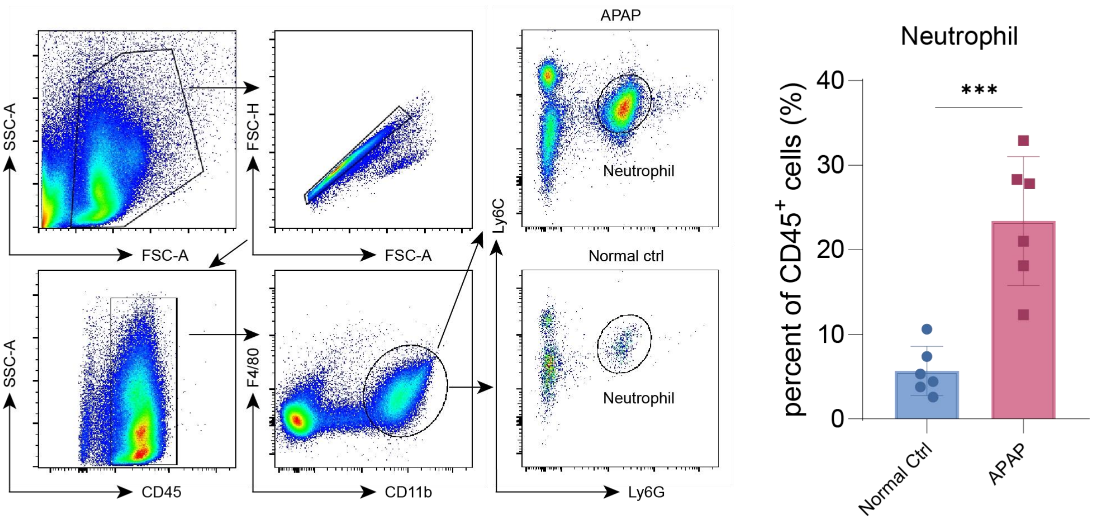

Figure S2. CD45+ neutrophil counts in the peripheral blood of the NC group and APAP-ACLF group were analyzed by flow cytometry (n = 5). *P < 0.05, **P < 0.01, ***P < 0.001, ***P < 0.0001.

Supplemental Figure 3:

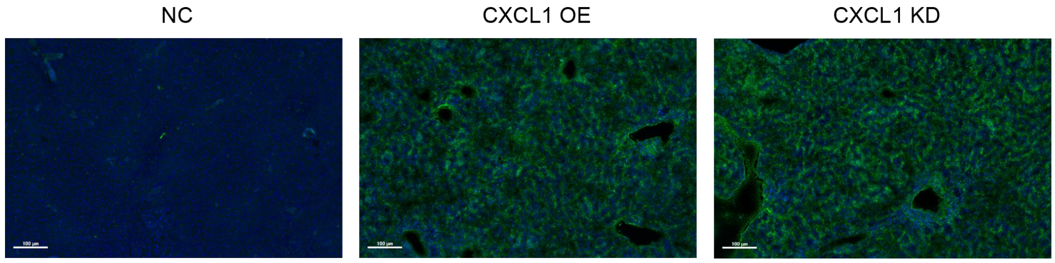

Figure S3. 28days after tail vein injection of AAV, the livers of mice in NC group, CXCL1 OE group and CXCL1 KD group were harvested, sectioned and photographed to observe the expression of GFP in the liver. Images representing the median expression levels of the group are shown. Bar = 100 μm.

Supplemental Figure 4:

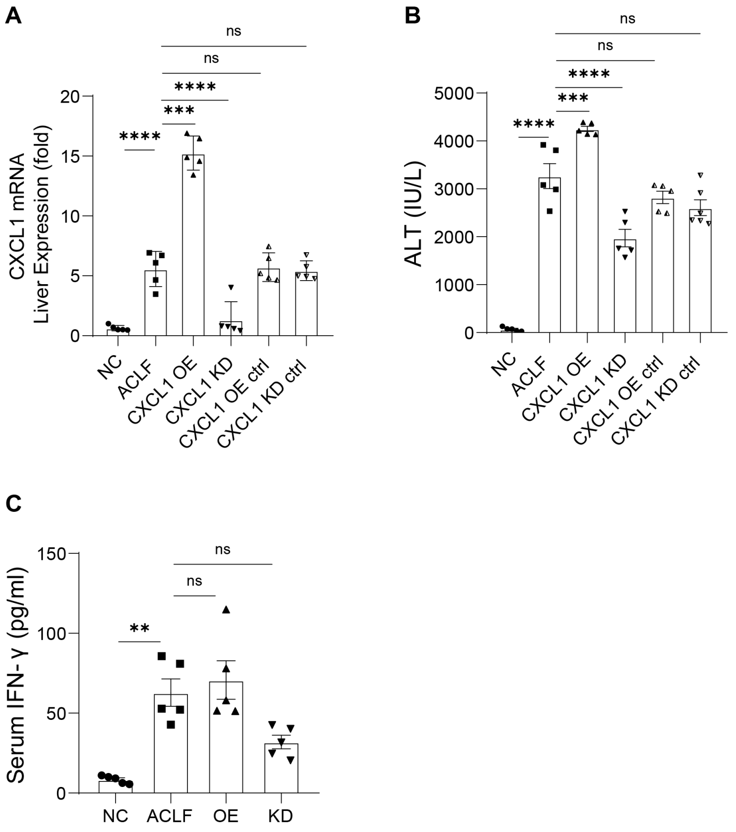

Figure S4. (A) Serum CXCL1 levels in each group were determined using ELISA (n = 5) ; The expression level of *Cxcl1* mRNA in the liver was determined using quantitative real-time reverse transcription PCR (n = 5). (B) Serum ALT level in each group (n =5). (C) Serum IFN- γ level in each group measured by ELISA (n = 5). (*P < 0.05, **P < 0.01, ***P < 0.001, ***P < 0.0001)

Supplemental Figure 5:

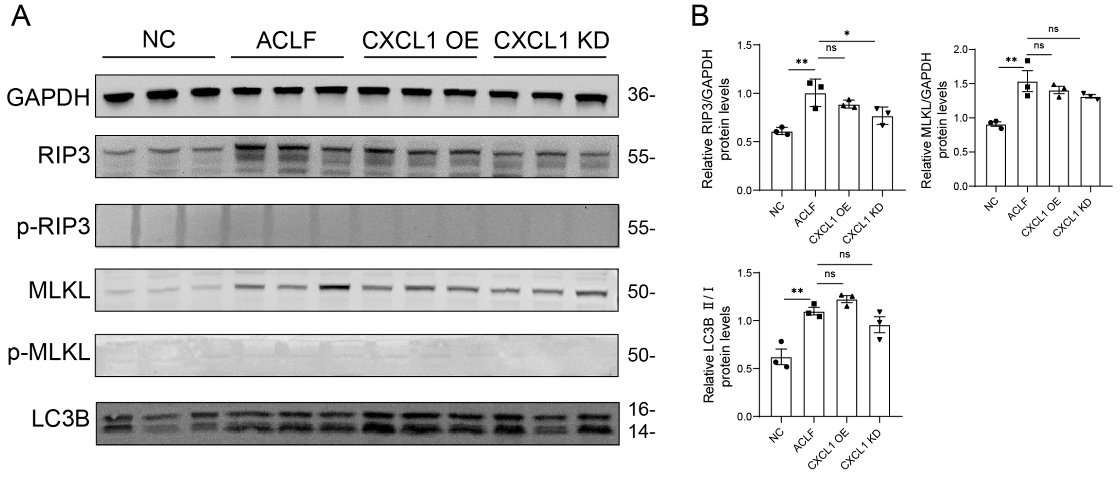

Figure S5. (A) Immunoblotting analysis of GAPDH, RIP3, p-RIP3, MLKL, p-MLKL and LC3B proteins in the liver. (B) Relative protein levels were measured using Image J (n=3). (*P < 0.05, **P < 0.01, ***P < 0.001, ***P < 0.0001)
